# 20 Years of Secretagogin: Exocytosis and Beyond

**DOI:** 10.3389/fnmol.2019.00029

**Published:** 2019-02-12

**Authors:** Magdalena Maj, Ludwig Wagner, Verena Tretter

**Affiliations:** ^1^Department of Biological Sciences, California Polytechnic State University, San Luis Obispo, CA, United States; ^2^Department of Internal Medicine III, Division of Nephrology and Dialysis, Medizinische Universität Wien, Vienna, Austria; ^3^Department of Anesthesia and General Intensive Care, Clinical Department of Anesthesia, Medizinische Universität Wien, Vienna, Austria

**Keywords:** secretagogin, calcium binding protein, calcium sensor, insulin secretion, hormone secretion

## Abstract

Calcium is one of the most important signaling factors in mammalian cells. Specific temporal and spatial calcium signals underlie fundamental processes such as cell growth, development, circadian rhythms, neurotransmission, hormonal actions and apoptosis. In order to translate calcium signals into cellular processes a vast number of proteins bind this ion with affinities from the nanomolar to millimolar range. Using classical biochemical methods an impressing number of calcium binding proteins (CBPs) have been discovered since the late 1960s, some of which are expressed ubiquitously, others are more restricted to specific cell types. In the nervous system expression patterns of different CBPs have been used to discern different neuronal cell populations, especially before advanced methods like single-cell transcriptomics and activity recording were available to define neuronal identity. However, understanding CBPs and their interacting proteins is still of central interest. The post-genomic era has coined the term “calciomics,” to describe a whole new research field, that engages in the identification and characterization of CBPs and their interactome. Secretagogin is a CBP, that was discovered 20 years ago in the pancreas. Consecutively it was found also in other organs including the nervous system, with characteristic expression patterns mostly forming cell clusters. Its regional expression and subcellular location together with the identification of protein interaction partners implicated, that secretagogin has a central role in hormone secretion. Meanwhile, with the help of modern proteomics a large number of actual and putative interacting proteins has been identified, that allow to anticipate a much more complex role of secretagogin in developing and adult neuronal cells. Here, we review recent findings that appear like puzzle stones of a greater picture.

## Introduction

Calcium ions (Ca^2+^) are one of the major messengers used by cells to regulate their metabolism, drive gene expression and activate specific cellular functions like exo- and endocytosis or contraction. In order to fulfill these tasks resting cells actively expel calcium ions from the cytosol with the help of Ca^2+^ transport ATPase (PMCA) and Na^+^/Ca^2+^ exchanger (NCX) that are localized in the plasma membrane and transport Ca^2+^ against a massive concentration gradient into the extracellular space (Rosado et al., 2006). Calcium is also stored intracellularly in the endoplasmatic reticulum (ER) or, as in specialized muscle cells, in the sarcoplasmic reticulum (SR) as well as in the mitochondria and is taken up into these compartments by the sarcoendoplasmic reticulum Ca^2+^ - ATPase (SERCA) or the mitochondrial Ca^2+^ uniporter. Activation of Ca^2+^-dependent signaling pathways can be initiated by membrane depolarization or extracellular signaling molecules that activate voltage- or ligand-activated calcium channels in the plasma membrane or *via* intracellular messengers that cause the release of Ca^2+^ from intracellular stores, mainly *via* the 1, 4, 5- triphosphate receptor (IP3R) or the ryanodine receptor (RyR) from the ER or SR (Supnet and Bezprozvanny, 2010). Beside these transporters and channels, cells can draw from a huge number of calcium-dependent interactions and signaling components, that Berridge et al. (2000) call the “Ca^2+^ signaling toolkit.” Parts of this universal toolkit are combined by individual cells to fulfill their special tasks. As Ca^2+^ flows into the cells due to so-called “ON mechanisms” it switches on multiple signaling cascades. The amplitude and duration of the signal is shaped by cytosolic Ca^2+^ buffers like parvalbumin, calbindin-D_28K_ and calretinin that transiently take up Ca^2+^ and contribute to the spatial restriction of the signal. This is especially important in neurons where compartmentalization of signaling is mandatory for neuronal transmission (Sudhof, 2004). In contrast to high-affinity Ca^2+^ buffers, lower affinity Ca^2+^ sensors respond to significant elevations of intracellular Ca^2+^ concentrations with conformational changes thereby facilitating interaction with downstream targets and initiating major cellular responses (Amici et al., 2009). Depending on the Ca^2+^ binding motifs there are three classes of Ca^2+^ binding proteins: the EF-hand proteins, the annexins and the C2 domain proteins (Bagur and Hajnoczky, 2017). The EF-hand is frequently found in buffers and sensors alike (structural vs. regulatory EF-hand) and contains Ca^2+^ in a complex with seven ligands in a loop flanked by two perpendicular α-helices (da Silva et al., 1995). Prominent members of this class are calmodulin, parvalbumin, troponin C, calretinin, calcineurin and secretagogin.

While the other representatives have been known for a long time and their function is well characterized, secretagogin’s precise role in specialized cell types has been beginning to emerge only recently. Here, we will review recent findings with regard to secretagogin’s cellular localization, interactions and functions as a novel Ca^2+^ sensor after a brief glance at the beginnings.

## Discovery of Secretagogin

Secretagogin (SCGN) was discovered in an “expression dictates function” manner. Wagner et al. (1998) generated the murine monoclonal antibody D24 (mAb D24) against an unknown human insulinoma-specific antigen, that binds to all cells present in the pancreatic islets of Langerhans and to all tested insulinomas but does not stain the exocrine pancreatic tissue. Screening of a human pancreatic beta cell cDNA library with D24 mAb identified a unique mRNA sequence, which encoded a novel Ca^2+^-binding protein related to human and murine calbindin and calretinin. A bioinformatic sequence analysis suggested that this novel protein has six tandem repeats of an EF-hand and a molecular weight of 32kDa. The newly discovered protein was named “Secretagogin” likely because it was shown to facilitate insulin release. Initial studies in Rin-5F rat insulinoma cells identified secretagogin mainly in the cytosolic fraction with some scant presence in the nuclei. Overexpression of secretagogin resulted in a greater calcium flux in response to KCl along with increased insulin secretion rates compared to vector controls (Wagner et al., 2000). Interestingly, these SCGN overexpressing Rin-5F clones had significantly lower cell growth rates. This fact correlated with down-regulation of substance-P (a putative β-cell trophic factor), known to be largely restricted to proliferating fetal and neonatal islet cells and to pancreatic beta cell lines. Therefore, it is possible that SCGN-induced suppression of substance-P transcription is responsible for the observed reduction in cell growth rates (Maj, 2012).

## Characteristics of Secretagogin and Interaction Network

Secretagogin is a hexa EF-hand protein that binds Ca^2+^ with an overall half maximal affinity of approximately 25 μM, a relatively low affinity compared to calbindin [with a (Ca^2+^) 0.5 of approx. 1 μM] or calretinin [with a (Ca^2+^) 0.5 of approx. 1.5 μM; Rogstam et al., 2007]. The secretagogin gene and protein structure has been reviewed in detail previously (Alpár et al., 2012).

The tertiary structure of secretagogin changes significantly upon Ca^2+^ binding, but not upon Mg^2+^ binding, suggesting that SCGN belongs to the “sensor” family of Ca^2+^ -binding proteins (Rogstam et al., 2007).

Expression of secretagogin can be influenced by insulin and glucose. In a cell culture study an insulin bolus was shown to increase secretagogin mRNA in hippocampal neurons after 24 h, while addition of glucose down-regulated SCGN mRNA levels already after 1 h (Maj et al., 2012). It has further been established, that SCGN mRNA expression in pancreatic islets negatively correlates with the incidence of diabetes and the levels of glycated hemoglobin in blood (Malenczyk et al., 2017). As a mechanism to activate secretagogin expression Malenczyk et al. (2017) could pinpoint the family of Ca^2+^permeable TRP(V) channels, that, when activated, induce nuclear translocation of the Sp1 transcription factor to initiate secretagogin transcription. Sp1 has previously been shown to also mediate upregulation of the CBP calmodulin in response to an insulin stimulus (Solomon et al., 1997).

A Ca^2+^ sensor responds to a strong Ca^2+^ signal with changes in protein conformation that can only be translated in cellular processes *via* interaction with other proteins. Therefore identification of protein interaction partners is crucial to understand functional mechanisms.

Rogstam et al. (2007) identified the first Ca^2+^ dependent interaction partner of SCGN by affinity purification from mouse and cow brain and the rat insulinoma cell line RIN-5F, which was identified as 25kDa synaptosome-associated protein (SNAP-25) in LC-MS/MS. SNAP-25 is one of the components of the soluble N-ethylmaleimide-sensitive-factor attachment receptor (SNARE; soluble N-ethylmaleimide-sensitive fusion protein attachment protein receptor) complex, which is the main complex for membrane fusion events (Chen and Scheller, 2001). As SNAP-25 is a protein involved in Ca^2+^-induced exocytosis in neurons and neuroendocrine cells, it was anticipated that secretagogin might be involved in the exocytosis processes of neurotransmitters and insulin granules.

Three years later nine other proteins were identified from screening a human protein array for Ca^2+^-dependent secretagogin interactors: SNAP-23, DOC2alpha, ARFGAP2, rootletin, KIF5B, β-tubulin, DDAH-2, ATP-synthase and myeloid leukemia factor 2 (Bauer et al., 2011). All of these interaction partners are involved in secretion events and vesicle trafficking. For example, SNAP-23 is structurally and functionally similiar to SNAP-25, binds to the SNAREs syntaxin and synaptobrevin and is an important regulator of transport vesicle docking and fusion. DOC2a itself is a Ca^2+^sensor, that promotes among others the spontaneous release of neurotransmitters glutamate and GABA (Courtney et al., 2018). ARFGAP2 is involved in specific cargo sorting to the plasma membrane. Kinesin 5B in neurons has been shown to be essential as motor protein in the anterograde transport of active zone proteins (Cai et al., 2007). Rootletin functions as a linker of the centrosome, which is the central microtubule organization center. Dimethylarginine dimethylaminohydrolase (DDAH) 2 is an enzyme, that metabolizes the NO-synthesis antagonist ADMA (asymmetric dimethylarginine). ATP synthase is an enzyme, that produces the cell’s main energy currency, ATP, which fires most cellular processes including intracellular transport. Tubulin as constituent of microtubuli is involved in major transport processes in the cell. Further, Ca^2+^-dependent interaction of secretagogin with the microtubule-associated protein (MAP) Tau was initially demonstrated by GST-pull downs from pancreatic cell lines and later confirmed from rat brains (Maj et al., 2010, 2012).

These SCGN interaction partners already implicate the possibility that secretagogin might link Ca^2+^ signaling to exocytotic processes.

In the last 3 years the discovered secretagogin interactome has grown extensively due to huge proteomic efforts. In Supplementary Table S1, we summarize to the best of our knowledge all published confirmed and putative interactors of secretagogin. Beside the pioneering studies, there are results from large scale proteomics essentially from two major groups available (Huttlin et al., 2015, 2017; Romanov et al., 2015; Hanics et al., 2017; Malenczyk et al., 2017, 2018). Without a special focus on secretagogin, Huttlin et al. (2015, 2017) found 16 putative interactions in their “BioPlex Network,” a high throughput combination of affinity purification with thousands of baits from a lentiviral library and mass spectrometry. Some of these hits were consistent with previously known proteins, indicating the validity of results.

Malenczyk et al. (2017, 2018) were specifically interested in SCGN interaction partners and used pull-down assays from pancreas and the INS-1E beta cell line combined with LC-MS/MS analysis. Hanics et al. (2017) focused on migrating SCGN^+^ neurons in the rostral migratory stream (RMS) and used micropunches from that area in co-immunoprecipitations. Similarily, Romanov et al. (2015) identified Ca^2+^-dependent and independent interactors by co-immunoprecipitations from hypothalamus using MALDI-TOF analysis.

**Figure 1:**
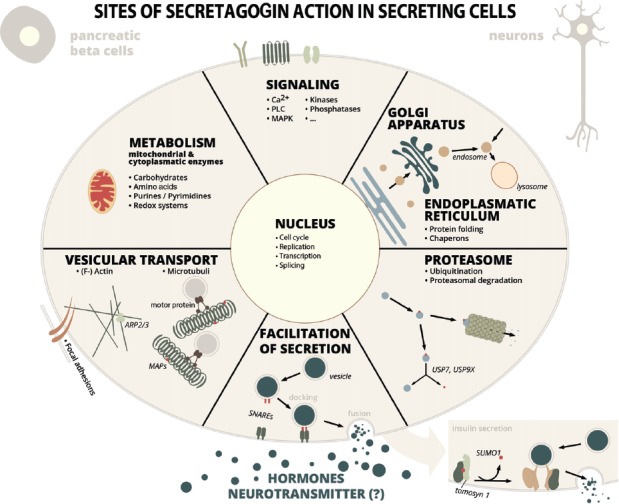
Sites of secretagogin action in secreting cells. Secretagogin action in secreting cells (insulin secreting pancreatic beta cells, stress hormone releasing neurons or other SCGN^+^ neurons) is manifold according to protein interaction partners identified by proteomic studies. Some of these processes have been investigated into detail (see main text for details), while the relevance of many other protein interactions are still unexplored. Abbreviations: MAP, microtubule-associated protein; SNARE, soluble N-ethylmaleimide-sensitive-factor attachment receptor; PLC, phospholipase C; MAPK, mitogen-activated protein kinase; Arp, actin-related protein; USP, ubiquitin-specific-processing protease.

The resulting interactome is extensive and can be functionally grouped into proteins involved in vesicle-mediated transport and exocytosis, protein folding, ubiquitination and proteasomal degradation, cytoskeleton-related proteins and their organization, carbohydrate metabolism, lipid metabolism, mitochondrial organization and processes, gene expression-related processes from DNA-replication to protein translation, metabolism of nucleotides, kinases and phosphatases and some other proteins (Supplementary Table S1, Figure 1). Certainly, not all interactions have been characterized in detail yet, but in many cases it can be anticipated, that these interactions are important for the function of secretory cells.

In their study Malenczyk et al. (2018) focused on proteins, that might have protective roles in supporting protein folding, stabilization and in preventing ER stress. Proteins like ubiquitin carboxyl-terminal hydrolases USP9X and USP7, that de-ubiquitinate their substrates and thereby stabilize them, are kept in an active form by secretagogin, in order to promote β-cell proliferation. Another example for such an interaction partner is T-complex protein 1 (CCT), a chaperonin, or ADP-ribosylation factor (Arf)4, involved in vesicle trafficking and a marker of ER/Golgi stress (Reiling et al., 2013), that is inversly regulated to secretagogin levels (Malenczyk et al., 2018). The cytosolic chaperonin CCT has an important role in the biogenesis of the actin cytoskeleton (Grantham et al., 2002) and was previously found to bind to chromaffin granules possibly involved in exocytosis (Creutz et al., 1994).

In order to elucidate more of secretagogin’s cellular functions, it is essential to note, that it is not only a pancreatic protein, but occurs in several other tissues, as it seems, frequently locally restricted to larger groups of cells. In this regard, secretagogin’s distribution in the CNS is of major interest.

## Cell Specific Distribution of Secretagogin in the Nervous System

Secretagogin has been studied in various organisms as it is expressed from zebrafish to human. Initial studies only roughly identified secretagogin positive areas in various species. Beside the pancreas, it was detected in the gastrointestinal tract, thyroid, adrenal medulla, adrenal gland and brain (Wagner et al., 2000). Especially prominent is SCGN in neuroendocrine cells such as the islet of Langerhans and subpopulations of developing or adult neurons (Gartner et al., 2010; Mulder et al., 2009, 2010).

However, secretagogin’s expression pattern is not conserved from rodents to humans and also significant differences exist between mice and rats (Garas et al., 2016; Raju et al., 2018). At the mRNA and protein level secretagogin has the highest expression in human cerebellum (Maj et al., 2012), while in mouse and rat it is found predominantly in the olfactory bulb (Mulder et al., 2009; Maj et al., 2012). Interestingly, SCGN is also highly expressed in the hippocampus of both, men and rodents. Immunohistochemical analysis identified secretagogin positive neurons mostly as interneurons and pyramidal cells (Gartner et al., 2010); SCGN is highly expressed in the molecular layer of human cerebellum, while in rat olfactory bulb it is found mainly in cell bodies of the granular layer and neurites and some cell bodies of the glomerular and external plexiform layer (Maj et al., 2012).

As mentioned before, it is apparent, that expression of secretagogin frequently occurs in cell clusters, shown for rat brain by Maj et al. (2012), implicating that these neurons either share common functional characteristics or their developmental origin. For example, SCGN^+^ clusters are seen in peripheral cell layers of the olfactory bulb, lining the surface above the optic nerve, in the supraoptic nuclei on both sides of the optic chiasm, in the suprachiasmatic nuclei of the hypothalamus, in the paraventricular nucleus (PVN), in the CA1-CA3 of the hippocampus, and outer cortex around the posterolateral cortical amygdaloid area. This specific expression of SCGN anticipates an important role in the mechanisms of olfaction, vision, activity of the hypothalamus, and memory formation in hippocampus.

Early immunocytochemical localization studies revealed that in addition to cytoplasmatic staining, SCGN seemed to be accumulated on specific subcellular structures in rat neurons. Specifically, SCGN^+^ clusters were often found in direct contact with GM130 clusters, a marker of the cis-Golgi network. Adding to the fact, ultracentrifugation experiments using sucrose density centrifugation of rat hippocampal tissue extracts revealed that SCGN immunoreactivity was detectable in the membrane-fraction as well as in the cytosolic fraction (Maj et al., 2012). In semiquantitative electron microscopy analyses of the PVN of the hypothalamus, gold particle-coupled secretagogin antibodies bound to the plasma membrane and to endomembranes even outnumbered cytosolic particles (Romanov et al., 2015).

More detailed studies aimed to characterize SCGN-positive cell populations with regard to co-expression of other cell markers are required. Gyengesi et al. (2013) investigated the SCGN expression pattern in neurons of the basal forebrain, the major cholinergic output of the central nervous system. SCGN is expressed in the forebrain, where Ca^2+^-dependent signaling plays an important role in neuronal plasticity and synaptic function. Disturbance of Ca^2+^ buffering capacity in the basal forebrain is observed to be increased in aged, cognitively impaired rats (Murchison et al., 2009). Hence, the study focused on characterizing cholinergic corticopedal neurons in the basal forebrain that play important roles in cortical activation, sensory processing, and attention. They demonstrated that SCGN is expressed in cell bodies of the medial and lateral septum, vertical and horizontal diagonal band nuclei and in the extension of the amygdala, but it is almost absent in the ventral pallidum. SCGN is co-localized with choline acetyltransferase (ChAT) in neurons of the bed nucleus of the stria terminalis, which is considered an important regulator site of the hypothalamic-pituitary-adrenal (HPA) axis, the extension of the amygdala, and the interstitial nucleus of the posterior limb of the anterior commissure. SCGN is frequently co-localized with calretinin, but not with parvalbumin or neuropeptide Y (Gyengesi et al., 2013).

A detailed characterization of SCGN-positive cells in zebrafish retina has been provided by Dudczig et al. (2017). SCGN starts to be expressed at day-3 postfertilization coinciding with the occurrence of Ca^2+^-waves initiated by retinal bipolar cells at a time, when larvae show first visual responses. SCGN stains a population of interneurons (approximately 60% GABA-ergic) in the inner nuclear layer with neurites extending into the inner plexiform layer. The expression of SCGN shows significant overlap with calbindin and calretinin, but not parvalbumin expression.

Secretagogin has been demonstrated to label several subtypes of cone bipolar cell in the mouse, rat, and rabbit retina (Puthussery et al., 2010). This finding was supported by a second study showing that SCGN is strongly expressed in the bipolar cell type DB1 in the macaque retina (Puthussery et al., 2011). Utilizing SCGN as a DB1 cell-specific marker allowed to investigate the cone connectivity as well as characterization of neurotransmitter receptors. This showed that the DB1 cells make synaptic contact with both L/M as well as S-cone photoreceptors and only minimal contact with rod photoreceptors (Puthussery et al., 2011).

In the human hippocampus, secretagogin occurs exclusively in CA1-CA4 and subiculum pyramidal neurons (Attems et al., 2007). Interneurons targeting striatal projection neurons were regarded as a relatively homogenous cell population due to their common expression of parvalbumin. However, co-staining for secretagogin has defined two novel subpopulations that preferentially innervate different pathways in the striatum (Garas et al., 2016). A very distinct population of SCGN-positive neurons with regard to other CBPs like calbindin or calretinin occurs in the habenula (Maj et al., 2012). Here, large groups of SCGN^+^ cells are flanked by similarily large groups of neurons expressing the other CBPs.

Secretagogin has also been found in a population of nociceptive dorsal root ganglia (DRG) neurons in the dorsal horn of mouse and human that coexpress calcitonin gene-related peptide (CGRP) and in rat DRGs not CGRP. SCGN-positive neurons were further found in the mouse, rat and human dorsal horn implicating a role of these neurons in processing of sensory information including pain (Shi et al., 2012).

An interesting fact is, that secretagogin is frequently expressed in young, not yet terminally differentiated neurons, like the migrating neuroblasts in the RMS and newly born dentate granule cells, possibly stimulating the release of factors necessary for the survival of these young neurons (Mulder et al., 2009). In the RMS of rodents, a large amount of newly born neuroblasts migrate towards the olfactory bulb to mediate sensory plasticity. Within the RMS a scaffold of wired secretagogin-positive neurons release on demand matrix-metalloprotease-2 (MMP-2) in order to loosen the extracellular matrix and to facilitate migration of the neuroblasts. MMP2-release is known to be mediated by annexin V, that has been identified as secretagogin interaction partner (see Supplementary Table S1).

Based on the unique SCGN expression and localization in the nervous system, this CBP broadens the repertoire of available cell-specific marker, which can be used in anatomical and neurophysiological studies, albeit with caution, as this population still might not be homogenous.

Meanwhile several studies have put effort in elucidating the mechanisms, how secretagogin actually functions in the cell. These have investigated its role in insulin release and have identified a neuronal correlate in hormone release.

## Secretagogin’s Role in Insulin Secretion

Early work already implicated that secretagogin influences insulin release (Wagner et al., 2000), while the detailed mechanism has only started to be understood recently. Glucose-stimulated insulin secretion (GSIS) is a multifactorial process that centrally involves an intracellular rise in Ca^2+^ concentration after the opening of voltage-dependent Ca^2+^ channels (Komatsu et al., 2013). The redox sensitive Ca^2+^ sensor secretagogin has been postulated to dimerize by forming a disulfide bridge in response to the concomitantly elevated ROS, thereby strengthening its association with the actin cytoskeleton (Yang et al., 2016; Lee et al., 2017). Secretagogin dissociates from the syntaxin1A-inhibitor tomosyn after its de-SUMOylation by the protease SENP1 and involves in insulin granule trafficking and exocytosis, *via* its other identified interaction partners, the t-SNAREs (Ferdaoussi et al., 2017). Secretagogin then assumingly gets involved in a complex of syntaxin and the v-SNARE VAMP2 in order to recruit insulin-laden vesicles to the plasma membrane. Finally, insulin release from the dense-core vesicles is regulated by synaptotagmin, a N-terminal transmembrane C2-domain Ca^2+^ sensor and v-SNARE, that also binds syntaxin and SNAP23/25 (MacDougall et al., 2018). However, secretagogin seems to have an additional function in restructuring the cytoskeleton during insulin release: it influences F-actin dynamics, in order to facilitate vesicle transport to the periphery, as well as focal adhesion remodeling (Yang et al., 2016). Proteomic studies have identified multiple SCGN-interacting proteins, that are either actin-binding proteins or have a regulatory function towards the actin cytoskeleton (see Supplementary Table S1).

## Control of Stress Hormone Release

A major finding of the last years has been the involvement of SCGN in the control of stress hormone release (Romanov et al., 2015). It has previously been described, that secretagogin occurs in groups of neurons in the hypothalamus, especially the PVN (Mulder et al., 2009; Maj et al., 2012). The PVN essentially contains two sorts of neurosecretory neurons: the magnocellular neurons secreting oxytocin and vasopressin in the posterior pituitary gland, and the parvocellular neurons secreting corticotropin-releasing hormone (CRH), vasopressin and thyrotropin-releasing hormone (TRH). Additionally, the PVN contains many neurons expressing neuropeptides. Calbindin-D_28k_ and calretinin mainly occur in magnocellular neurosecretory cells (Sánchez et al., 1992; Arai et al., 1994), while secretagogin antibodies predominately stain a subpopulation of parvocellular neurons in the dorsolateral PVN. In their study secretagogin mRNA mainly occured together with transcripts for CRH, somatostatin, tachykinin 1, amphetamin-regulated transcript, neuromedin B, neuromedin U, neuropeptide Y and neuropeptide B, but not with vasopressin, oxytocin, thyrotropin-releasing hormone (TRH), galanin, cholecystokinin, neurotensin S, calcitonin, neuromedin S, natriuretic peptide C and adenylate cyclase activating polypetide 1. Axons of the parvocellular neurosecretory neurons project to the median eminence where they release their hormones to the hypophyseal portal system. On the ultrastructural level they found, that secretagogin not only occurs in the cytoplasm and close to the ER, but also in axonal terminals, frequently associated with the plasma membrane and the outer membrane of dense-core vesicles containing CRH implicating that it might be involved in CRH release. Indeed, knock-down of secretagogin reduced CRH release from PVN neurons, similiary like insulin release from pancreatic β-cells. *in vivo* knock-down of secretagogin by injection of siRNA into the lateral ventricle led to hypothalamic SCGN downregulation by 20–30% concomitant with CRH retention in the soma of parvocellular neurons. These results gain even more significance, as the authors were able to prove, that secretagogin plays a significant role in the overall stress response. CRH function is to stimulate the release of adenocorticotropic hormone (ACTH) from the anterior pituitary gland and ACTH then activates the adrenal glands to release steroid hormones like cortisol, the primary stress hormone. Romanov et al. (2015) could show in an *in vivo* stress model, that the acute stress led to the activation of the immediate early gene c-fos mostly in secretagogin-positive neurons of the PVN and the corresponding ACTH response including corticosterone levels in the blood could be blunted by secretagogin siRNA-pretreated animals.

Authors from the same group went on to find out, that CRH neurons not only mediate HPA-axis activation, but also influence cortical excitability in a secretagogin-dependent fashion. The prefrontal cortex (PFC) coordinates vigilance, alertness and behavioral responses to acute stress facilitated by noradrenergic afferents originating in the locus coeruleus. These norepinephrinergic (NE^+^) cells on one hand are directly innervated by CRH^+^ cells (Zhang et al., 2017), but on the other hand inter-neuronal communication is bypassed by volume transmission from ependymal cells *via* the cerebrospinal fluid in order to extend responses over longer periods. Specifically, CRH^+^ cells from PVN also innervate ependymal cells of the 3rd ventricle inducing ciliary neurotrophic factor (CNTF) release into the cerebrospinal fluid. In the locus coeruleus, CNTF binds to its cognate Trk receptor on NE^+^ neurons initiating a signaling cascade of ERK1 activation (a secretagogin-interactor) and subsequent tyrosine hydroxylase (TH) phosphorylation thereby stimulating its enzymatic activity. Alpár et al. (2018) proved a central role of secretagogin in mediating TH-phosphorylation in knock-down experiments and observed a blunted behavioral stress response in secretagogin knock-out animals. A detailed mechanism of secretagogin action in this context is still missing, however the necessity of secretagogin presence for proper signal transduction in the hypothalamus-locus coeruleus-prefrontal cortex circuit of stress response is obvious.

The data presented by Romanov et al. (2015) and Alpár et al. (2018) define a critical role of secretagogin in the molecular axis of stress responsiveness. Their results let the study of Gyengesi et al. (2013) appear in a new light: the bed nucleus of the stria terminalis functions as a regulator of the HPA-axis and many of the SCGN-positive neurons identified in this area are CRH-positive. Similarily, extrahypothalamic CRH also affects neuronal firing in the lateral habenula, a brain region that negatively regulates the dopamine-dependent reward circuit and mediates emotional responses to stress (Authement et al., 2018). Secretagogin is highly expressed in this area, as described above. However further investigations into SCGN function in this area of the brain have not been performed yet.

Dysfunction of the HPA-axis is associated with pathological developments as observed in Alzheimer’s disease (AD), major depression, and serious metabolic consequences including hypoglycemia.

## Secretagogin in Disorders and Diseases of the CNS

The crucial roles of secretagogin in cellular function as defined by today already put it into an important position in the context of pathological developments.

Clinical data have suggested links between Type II diabetes (T2D) and AD, which some researchers have proposed to call a form of “Type III diabetes.” Animal models have shown that T2D promotes the formation of amyloid-β plaques, tau phosphorylation and neurofibrillary lesions, however in humans this evidence is scarce or ambigous. What has been shown, is that brain insulin resistance with an impaired signaling response to insulin is also a pathomechanism in AD (Arnold et al., 2018).

It is interesting to note that secretagogin’s main expression in the brain occurs in regions with the highest density of insulin receptor, like the olfactory bulb, hypothalamus, hippocampus, cortex, striatum and cerebellum. *De novo* insulin production in the brain has been proven, but still needs further investigation. However, insulin—either secreted from neurons or entered into the brain *via* the blood-brain barrier—has enormous impact on neuronal function like trafficking of neurotransmitter receptors, synaptic plasticity, synapse formation and neuronal survival. It can only be speculated that secretagogin could have a role in neuronal insulin secretion.

In addition to its role in insulin secretion it has been discussed whether secretagogin expressing neurons could be protected from pathological manifestations and cell death in diseases like AD. It has been shown, that CBPs like parvalbumin, calbindin-D_28K_ and calretinin (i.e., Ca^2+^ buffers) protect neurons from detrimental Ca^2+^ overload as it occurs for instance in ischemic conditions (Turovsky et al., 2018). Although secretagogin is considered to be mainly a Ca^2+^ sensor, it also has one high-affinity Ca^2+^ binding site and could also have some buffering capacity (Rogstam et al., 2007). With this regard, Khandelwal et al. (2017) have shown, that the Ca^2+^ affinity of secretagogin also depends on the redox-status of the protein. Under reducing conditions the overall affinity for Ca^2+^ significantly increases to the nM range.

Dysregulated Ca^2+^ signaling seems also to underlie neuronal dysfunction in the development of AD (Magi et al., 2016). Key manifestations of the disease are amyloid plaques and neurofibrillary tangles from hyperphosphorylated tau protein. Post mortem studies found that colocalization of secretagogin and hyperphosphorylated tau was negligable in brains from patients with AD of Braak stage III or higher and that the number of secretagogin-positive cells was not significantly changed in brains with different tau burden (Attems et al., 2008). These findings supported the hypothesis that secretagogin-positive neurons are possibly resistant to neurodegeneration at least in this area of the brain. However, Attems et al. (2011) in a later study investigated secretagogin expression in the brains of P301L tau transgenic mice, a mouse model for tau pathology in AD. They found significant reduction of secretagogin expression in P301L homozygous mice.

A special situation obviously exists in the olfactory system of humans and primates. Here, Attems et al. (2012) identified a population of not terminally differentiated, yet synaptically integrated secretagogin-positive neurons in the olfactory tract of humans, that are selectively affected by neurofibrillary pathology in AD and seem to be lost in advanced stages of the disease. Perturbed olfaction is an early clinical sign in AD and this might involve the loss of these special shell cells. However, secretagogin-positive cells in the periglomerular layer seemed not to be affected.

A possible beneficial role of secretagogin was postulated in multiple sclerosis therapy. Patients with multiple sclerosis are recommended to take a dietary supplementation of vitamin D. However, the exact molecular mechanism behind the beneficial role of vitamin D are not well-known. Oveland et al., 2018 have recently shown that a high dose of the hormonally active 1, 25-dihydroxyvitamin-D3 (1, 25D) promotes myelin repair in the cuprizone model for de- and remyelination. They studied the brain proteome in the cuprizone model for de- and remyelination and found that 125 proteins were differentially regulated in brain tissue from 1, 25D-treated mice during remyelination, compared to placebo. Proteins upregulated in the early remyelination phase were involved in calcium binding, e.g., calretinin, S10A5 and secretagogin. Oveland et al., 2018 concluded that vitamin D may influence remyelination by mechanisms involving an increase in CBPs including secretagogin.

Although secretagogin is not co-released with glucose-stimulated insulin, it is found in plasma and concentrations are elevated in T2D patients (Hansson et al., 2018). In their *in vitro* studies in human β-cells they found increased secretagogin release due to ER stress inducers or due to inflammatory cytokines, implicating higher plasma levels as a biomarker for islet dysfunction.

Secretagogin has also been proposed as a biomarker for tumors and in ischemic brain insults, i.e., stroke. However, its plasma concentrations were either found lower or higher than in controls, dependent on the time of blood sampling (Gartner et al., 2010; Montaner et al., 2011).

Another study demonstrated that SCGN plasma levels were significantly lower in autistic children as compared to the healthy controls (Alhowikan et al., 2017). Children with severe and mild to moderate autism had significantly lower SCGN levels than healthy controls, however, there was no significant difference between the severity of autism and SCGN levels. In schizophrenia, decreased levels of SCGN mRNA were measured in post-mortem pituitaries, as well as in serum from a cohort of living first-onset schizophrenia patients (Krishnamurthy et al., 2013).

These studies convey an insight in the complex roles of SCGN in the neuronal network, specifically in aging, neurodegeneration and neuro-psychiatric disorders. Future studies will have to pave the way for translation of novel diagnostics and to investigate, if SCGN can serve as a biomarker especially in neuro-psychiatric onsets.

## Conclusion and Outlook

Secretagogin’s role as a Ca^2+^ sensor is now well established from the available experimental data. Its location and interaction with proteins involved with vesicle trafficking and in the exocytotic machinery not only implicates involvement in the release of signaling molecules, but this function has already been proven for insulin release from pancreatic β-cells and the stress response in the brain (CRH-release from parvocellular neurons of the PVN in the hypothalamus and activation of norepinephrinergic neurons in the locus coeruleus). Future research will prove, if secretagogin is engaged in the release of further hormones, or eventually even neuropeptides or other neurotransmitters. With this aim in mind, single-cell transcriptomics can be a suitable methodology for expression profiling of SCGN^+^ cells. However secretagogin’s protein interaction network, as known by today, is vast. Therefore it can be assumed, that in those selected cell types, where it is expressed, it might function as a kind of Ca^2+^-dependent switch or moderator for more cellular processes including cytoskeletal rearrangements and modulation of cellular stress. In order to delineate individual mechanisms, further functional investigations with known interactors will have to be undertaken.

## Author Contributions

All authors were involved in the conception of the article, MM and VT wrote the final version of the manuscript.

## Conflict of Interest Statement

The authors declare that the research was conducted in the absence of any commercial or financial relationships that could be construed as a potential conflict of interest.
